# Supplementary material for: Patterns of mobility and its impact on retention in care among people living with HIV in the Manhiça District, Mozambique
Source: PLoS One. 2021 May 21;16(5):e0250844. doi: 10.1371/journal.pone.0250844 (PMC8139482; doi:10.1371/journal.pone.0250844)
Supplement: S1 File — (DOCX) [file pone.0250844.s001.docx]

| 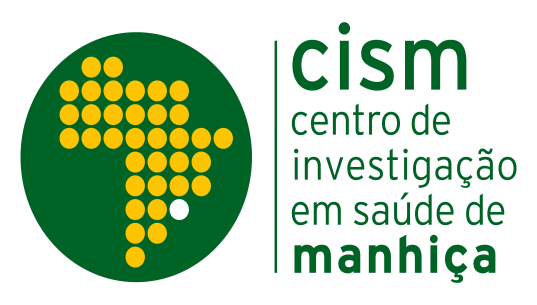 | **Study: DIASPORA**  **Survey: MOBILE_ADULT** | Serial number |
| --- | --- | --- |

|  | 1. **Does patiente lives currently in Manhiça District (DM)** ם Yes ם No 2. If participant leaves in MD, where?   □ Vila da Manhiça □ Maragra □ Palmeira/Nwamatibjana  □ Xinavane □ Maluana □ Taninga  □ Munguini □ Calanga □ 3 Fevereiro  □ Ilha Josina □ Xibukutsu □ Malavel  □ Outro \|__\|__\|__\|__\|__\|__\|__\|__\|__\|__\|__\|  2.1 If participant lives in DM,did change residency in the last 12 months?  ם Yes ם No ם Do not Know  2.2 If participant changed residency, was within Manhiça District?  ם Yes ם No ם Do not Know  2.3 If answer for question 2.2 is No, where to?  ☐Another district within Mozambique ☐ Another Country  2.4. If changed residency for another district within the country, which one?  \|__\|__\|__\|__\|__\|__\|__\|__\|__\|__\|__\|__\|__\|__\|__\|__\|__\|__\|__\|__\|__\|__\|__\|__\|__\|__\|__\|__\|__\|  2.5. If changed to another country, which one?  ☐ South Africa ☐ Swazilând ☐ Lesotho ☐ Zimbabwe ☐ Tanzânia ☐ Botswana  ☐ Other \|__\|__\|__\|__\|__\|__\|__\|__\|__\|__\|__\|__\|__\|__\|__\| | | | | | |
| --- | --- | --- | --- | --- | --- | --- |
|  | | **Full Name** | | \|__\|__\|__\|__\|__\|__\|__\|__\|__\|__\|__\|__\|__\|__\|__\|__\|  \|__\|__\|__\|__\|__\|__\|__\|__\|__\|__\|__\|__\|__\|__\|__\|__\|  \|__\|__\|__\|__\|__\|__\|__\|__\|__\|__\|__\|__\|__\|__\|__\|__\| | | |
|  | | **Head of Household Name** | \|__\|__\|__\|__\|__\|__\|__\|__\|__\|__\|__\|__\|__\|__\|__\|__\|  \|__\|__\|__\|__\|__\|__\|__\|__\|__\|__\|__\|__\|__\|__\|__\|__\|  \|__\|__\|__\|__\|__\|__\|__\|__\|__\|__\|__\|__\|__\|__\|__\|__\| | | | |
|  | | **Neighborhood** \|__\|__\|__\|__\|__\|__\|__\|__\|__\|__\|__\|__\|__\|__\|__\|__\| | | | | |
|  | | **Date of Birthy** | | | | \|__\|__\| - \|__\|__\|__\| - \|__\|__\|__\|__\| |
|  | | **Maritual Status** ☐ Married or living with a partner | | | | ☐ Separeted ☐ Widowed ☐ Single |
|  | | **Participant Perm_id** | | | \|__\|__\|__\|__\|- \|__\|__\|__\|-\|__\|__\| | |

**DIASPORA Participant identification**

**Mobile Recruted adult**

Mobile History

To fullfil if patient changed residency

9. How many times travelled outside Manhiça in the last year?

☐ 1-2 ☐3-4 ☐ >5

10. Destination place?

☐ Rural/countryside ☐ Urban/City ☐ Different places ☐ Do not Know

11. How long stayed at destination place?

☐ Less than 15 days ☐ From 15 days to 3 months ☐ From 3 to 12 months ☐ More than 12 months

☐ Dont know

12. How many times returned to Manhiça residency in the last year?

☐ Once a year ☐ From 6 and 6 months ☐ From 3 and 3 months ☐ Once a month

☐ Once a week ☐ Do not Know

☐ Outro |__|__|__|__|__|__|__|__|__|__|__|__|__|__|__|__|

13. How long stays at residency when return

☐ More than a month ☐ Froma week to one month ☐ Less than a week

☐ Do not Know ☐ Other |__|__|__|__|__|__|__|__|__|__|__|__|__|__|__|__|

14. Received any visit from family members while outside Manhiça?

☐ Yes ☐ No ☐ Do not Know

14.1. If yes, how many times in the last year?

☐ 1-2 ☐3-4 ☐ >5

15. Travelled with someone to destination? (check all that apply)

☐ Childrens ☐ Partner ☐ Parents ☐ Other family member ☐ No one

☐ Don’t Know ☐ Other |__|__|__|__|__|__|__|__|__|__|__|__|__|__|__|__|

16. What was the reason of changing residency?

☐ Work ☐ Study ☐ Marriage ☐ Health ☐ Seeking better opportunities

☐ Outro |__|__|__|__|__|__|__|__|__|__|__|__|__|__|__|__|

17. What kind of job did at destiny?

☐ Farm ☐ Industry ☐ Mine ☐ seller ☐ Domestic work

☐ Don’t work ☐ Other |__|__|__|__|__|__|__|__|__|__|__|__|__|__|__|__|

17.1.1. If miner in which province/region work?

|__|__|__|__|__|__|__|__|__|__|__|__|__|__|

17.1.2. If miner, in which kind of mine?

☐ Gold ☐ Coal ☐ Platin ☐ Diamants

☐ Other |__|__|__|__|__|__|__|__|__|__|__|__|__|__|__|__|

17.2 If farmer, in which province/region work?

|__|__|__|__|__|__|__|__|__|__|__|__|__|__|

18. As a passport? ☐ Yes ☐ No ☐ Do not Know

19. As a work visa? ☐ Yes ☐ No ☐ Do not Know

☐ Other |__|__|__|__|__|__|__|__|__|__|__|__|__|__|

20. What kind of accomodation at destiny?

☐ Family Home ☐ Work house ☐ own house ☐ room/rent house

☐ Other |__|__|__|__|__|__|__|__|__|__|__|__|__|__|

21. How many cellphones as?

☐ Non ☐ 1 ☐ 2-4 ☐ >5

21.1 If answer to question 2.is another country, have cellphone at destination?

☐ Yes ☐ No ☐ Do not Know ☐ Not Applicable

22. Communicates with family throw cellphone when at destination?

☐ Yes ☐ No ☐ Do not Know

HIV History

23. Remember when was diagnosed with HIV? ☐ Yes ☐ No

23.1. If Yes, when?

☐ Less than a year ☐ More than a year ☐ Do not Know

23.2. Changed residency before diagnose? ☐ Yes ☐ No ☐ Do not Know

24. Chaged residency before initiate ARV treatment? ☐ Yes ☐ No ☐ Do not Know

25. Since initiated follow up at HIV Care, homw many times changed residency?

☐ 1-2 ☐3-4 ☐ >5 ☐ Do not Know

26. Since changed residency continued HIV care follow up at Manhiça District?

☐ Yes ☐ No ☐ Do not Know

26.1. If not, was transfered for HIV care follow up at destination?

☐ Yes ☐ No ☐ Do not Know

26.2. If transference wasn’t done, what was the reason?

☐ Did not inform changing residency ☐ Requested but wasn’t accepted ☐ Do not Know

☐ Other |__|__|__|__|__|__|__|__|__|__|__|__|__|__|__|__|__|__|__|__|__|__|__|__|__|__|

|__|__|__|__|__|__|__|__|__|__|__|__|__|__|__|__|__|__|__|__|__|__|__|__|__|__|

27. Did HIV care follow up at destination? ☐ Yes ☐ No ☐ Do not Know

28. Had access to ARV at destination? (If No go to 28.1, if Yes go to 28.2)

☐ Yes ☐ No ☐ Do not Know

28.1. If not, what was the reason?

☐ Did not look for health care

☐ Did nott know that could follow up at destination

☐ Follow up was not accepted at destination

☐ ARV out of stock

☐ Do not know

☐ Other |__|__|__|__|__|__|__|__|__|__|__|__|__|__|__|__|__|__|__|__|__|__|__|__|__|__|

|__|__|__|__|__|__|__|__|__|__|__|__|__|__|__|__|__|__|__|__|__|__|__|__|__|__|

28.2. If yes, how access to ARV at destination?

☐ Sent by family/someone familiar ☐ Local pharmacy ☐ Bought it in a market/shop

☐ Other |__|__|__|__|__|__|__|__|__|__|__|__|__|__|__|__|__|__|__|__|__|__|__|__|__|__|__|

29. During your absence, someone picked up ARV for you at Manhiça District Health facility?

☐ Yes ☐ No ☐ Do not Know

29.1. If someone picked up, who was it? (check all that apply)

☐ Partner ☐ Children ☐ Parents ☐ Confident

☐ Other family member ☐ GAAC member ☐ Do not Know

☐ outro |__|__|__|__|__|__|__|__|__|__|__|__|__|__|__|__|

29.2. If someone picked up, how did it reach you?

☐ Mail ☐ Intermediary ☐ Other |__|__|__|__|__|__|__|__|__|__|__|__|__|__|__|__|

29.3. ARV got to you on time? ☐ Yes ☐ No ☐ Don’t Know

29.4. What you used to do without getting ARV’s from Manhiça?

☐ Look for ARV at destination health facility?

☐ Buy ARV’s at destination.

☐ Take less ARV’s until get other ones.

☐ Stop treatement until get othe rones

☐ Do not know

☐ Other |__|__|__|__|__|__|__|__|__|__|__|__|__|__|__|__|__|__|__|__|__|__|__|__|__|__|

|__|__|__|__|__|__|__|__|__|__|__|__|__|__|__|__|__|__|__|__|__|__|__|__|__|__|

|__|__|__|__|__|__|__|__|__|__|__|__|__|__|__|__|__|__|__|__|__|__|__|__|__|__|

30. Was anytime sick at destination? ☐ Yes ☐ No ☐ Do not Know

31. Anytime looked for health care at destination?

☐ Yes ☐ No ☐ Do not know

31.1. If answer for previous question was yes, where?

☐ emergency room ☐ Hiv care consultation ☐ prenatal consultation ☐ screening

☐ Do not know ☐ Other |__|__|__|__|__|__|__|__|__|__|__|__|__|__|__|__|

32.Was hospitalized at destination? ☐ Yes ☐ No ☐ Do not know

33. When return to Manhiça can easily return to HIV care consultation?

☐ Yes ☐ No ☐ Do not know

34. Did you ever stopped ARV treatment? ☐ Yes ☐ No ☐ Don’t know

34.1. If answer for previous question was Yes, for how long?

☐ Less than a month ☐ From one to 3 months ☐ More than 3 months ☐ Do not Know

35. Did you ever checked for CD4 and Viral Load at destination?

☐ Yes ☐ No ☐ Do not Know

36. If it was possible, would like to communicate with Manhiça District Health Facility at destination, by Text message?

☐ Yes ☐ No ☐ Do not know

Social Factors, Risk Factors

37. Do you have childrens? ☐ Yes ☐ No ☐ Do not Know

37.1. If have childrens, how many? |__|__|

38. Do you use condoms with your regular partner?

☐ Always ☐ No ☐ Not always ☐ Do not Know

39. Your partner knows about you HIV status?

☐ Yes ☐ No ☐ Do not Know

40. How many casual partners had in the last year? |__|__|__|

41. In your casual relationships did you use condoms?

☐ Always ☐ No ☐ Not Always ☐ Do not Know

42.Do you have another relationship at destination?

☐ Yes ☐ No ☐ Do not Know

43. Do you have childrens at destination? ☐ Yes ☐ No ☐ Do not Know

44. Do you use one of the followings? (check if apply)

☐ Alcohol ☐ Cigar ☐ Cannabis ☐ Do not Know

☐ Other |__|__|__|__|__|__|__|__|__|__|__|__|__|__|__|__|
