# Supplementary material for: Patterns of mobility and its impact on retention in care among people living with HIV in the Manhiça District, Mozambique
Source: PLoS One. 2021 May 21;16(5):e0250844. doi: 10.1371/journal.pone.0250844 (PMC8139482; doi:10.1371/journal.pone.0250844)
Supplement: S2 File — (DOCX) [file pone.0250844.s002.docx]

| 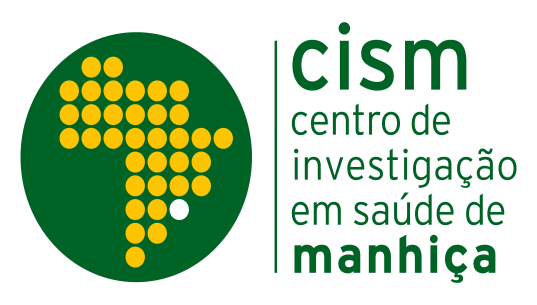 | **Estudo: DIASPORA**  **Inquérito: ADULTO_ MIGRANTE** | Serial number |
| --- | --- | --- |

|  | 1. **Atualmente, o participante vive no Distrito da Manhiça (DM)** ם Sim ם Não 2. Se o participante vive no DM, aonde?   □ Vila da Manhiça □ Maragra □ Palmeira/Nwamatibjana  □ Xinavane □ Maluana □ Taninga  □ Munguini □ Calanga □ 3 Fevereiro  □ Ilha Josina □ Xibukutsu □ Malavel  □ Outro \|__\|__\|__\|__\|__\|__\|__\|__\|__\|__\|__\|  2.1 Se o participante vive no DM, mudou de residência nos últimos 12 meses?  ם Sim ם Não ם Não sabe  2.2 Se o participante mudou de residência, foi dentro do próprio distrito da Manhiça?  ם Sim ם Não ם Não sabe  2.3 Se a pergunta 2.2 é NAO, para onde se mudou?  ☐ Outro distrito dentro de Moçambique ☐ Outro país  2.4. Si mudou para outro distrito dentro de Moçambique, onde?  \|__\|__\|__\|__\|__\|__\|__\|__\|__\|__\|__\|__\|__\|__\|__\|__\|__\|__\|__\|__\|__\|__\|__\|__\|__\|__\|__\|__\|__\|  2.5. Si mudou para outro país, para qual?  ☐ África do Sul ☐ Suazilândia ☐ Lesoto ☐ Zimbabwe ☐ Tanzânia ☐ Botswana  ☐ outro \|__\|__\|__\|__\|__\|__\|__\|__\|__\|__\|__\|__\|__\|__\|__\| | | | | | |
| --- | --- | --- | --- | --- | --- | --- |
|  | | **Nome completo** | | \|__\|__\|__\|__\|__\|__\|__\|__\|__\|__\|__\|__\|__\|__\|__\|__\|  \|__\|__\|__\|__\|__\|__\|__\|__\|__\|__\|__\|__\|__\|__\|__\|__\|  \|__\|__\|__\|__\|__\|__\|__\|__\|__\|__\|__\|__\|__\|__\|__\|__\| | | |
|  | | **Nome Chefe agregado** | \|__\|__\|__\|__\|__\|__\|__\|__\|__\|__\|__\|__\|__\|__\|__\|__\|  \|__\|__\|__\|__\|__\|__\|__\|__\|__\|__\|__\|__\|__\|__\|__\|__\|  \|__\|__\|__\|__\|__\|__\|__\|__\|__\|__\|__\|__\|__\|__\|__\|__\| | | | |
|  | | **Bairro em que vive** \|__\|__\|__\|__\|__\|__\|__\|__\|__\|__\|__\|__\|__\|__\|__\|__\| | | | | |
|  | | **Data de nascimento** | | | | \|__\|__\| - \|__\|__\|__\| - \|__\|__\|__\|__\| |
|  | | **Estado civil** ☐ Casado/a ou vivendo maritalmente | | | | ☐ Separado/a ☐ Viúvo/a ☐ Solteiro/a |
|  | | **Perm_id do participante** | | | \|__\|__\|__\|__\|- \|__\|__\|__\|-\|__\|__\| | |

**Identificação do participante DIASPORA**

**ADULTOS RECRUTADOS COMO MIGRANTES**

HISTÓRIA DE MIGRAÇAO

A preencher se o paciente mudou de residência.

9. Quantas vezes viajou fora de Manhiça no ultimo ano?

☐ 1-2 ☐3-4 ☐ >5

10. O lugar de destino foi?

☐ Rural/Campo ☐ Urbano/cidade ☐ Ha varios lugares de destino ☐ Não sabe

11. O senhor/senhora ficou por quanto tempo no lugar de destino?

☐ Menos de 15 dias ☐ De 15 dias à 3 meses ☐ De 3 à 12 meses ☐ Mais de 12 meses

☐ Não sabe

12. Quantas vezes voltou para a sua residência em Manhiça no último ano?

☐ Uma vez por ano ☐ De 6 em 6 meses ☐ De 3 em 3 meses ☐ Uma vez por mês

☐ Uma vez por semana ☐ Não sabe

☐ Outro |__|__|__|__|__|__|__|__|__|__|__|__|__|__|__|__|

13. Quanto tempo fica em casa quando volta?

☐ Mais de um mês ☐ De uma semana à um mês ☐ Menos de uma semana

☐ Não sabe ☐ Outro |__|__|__|__|__|__|__|__|__|__|__|__|__|__|__|__|

14. Foi visitado por alguém da sua família no tempo em que esteve fora da Manhiça?

☐ Sim ☐ Não ☐ Não sabe

14.1. Se sim, quantas vezes foi visitado no último ano?

☐ 1-2 ☐3-4 ☐ >5

15. Foi acompanhado por alguém ao lugar de destino? (assinale todas aplicáveis)

☐ Filhos ☐ Parceiro ☐ Mãe/ pai ☐ Outro familiar ☐ Ninguém

☐ Não sabe ☐ Outro |__|__|__|__|__|__|__|__|__|__|__|__|__|__|__|__|

16. Qual foi o motivo da mudança de residência?

☐ Trabalho ☐ Estudos ☐ Casamento ☐ Saúde ☐ Procura de melhores condições de vida

☐ Outro |__|__|__|__|__|__|__|__|__|__|__|__|__|__|__|__|

17. Que tipo de trabalho fez no lugar de destino?

☐ Agricultura ☐ Industria ☐ Mineiro ☐ Vendedor ☐ Trabalho domestico

☐ Não trabalha ☐ Outro |__|__|__|__|__|__|__|__|__|__|__|__|__|__|__|__|

17.1.1. Se mineiro, em que província/ região trabalha?

|__|__|__|__|__|__|__|__|__|__|__|__|__|__|

17.1.2. Se mineiro, em que tipo de mina?

☐ Oro ☐ Carvão ☐ Platina ☐ Diamantes

☐ Outro |__|__|__|__|__|__|__|__|__|__|__|__|__|__|__|__|

17.2 Se é agricultor, em que província/ região trabalha?

|__|__|__|__|__|__|__|__|__|__|__|__|__|__|

18. O senhor/a senhora tem passaporte? ☐ Sim ☐ Não ☐ Não sabe

19. O senhor/a senhora tem visto de trabalho? ☐ Sim ☐ Não ☐ Não sabe

☐ Outro |__|__|__|__|__|__|__|__|__|__|__|__|__|__|

20. Qual é o tipo de alojamento no lugar de destino?

☐ Casa familiar ☐ Casa da empresa ☐ Casa própria ☐ Quarto/ casa alugado

☐ Outro |__|__|__|__|__|__|__|__|__|__|__|__|__|__|

21. Quantos telemoveis celulares o senhor/senhora tem?

☐ Nenhum ☐ 1 ☐ 2-4 ☐ >5

21.1 Se a resposta a pergunta 2.5 é outro pais, o senhor/senhora tem telemovel do pais do destino?

☐ Sim ☐ Não ☐ Não sabe ☐ Não aplicavel

22. Comunica-se com a familia através do telefone quando esta no lugar de destino?

☐ Sim ☐ Não ☐ Não sabe

HISTORIA HIV

23. Lembra-se de quando foi diagnosticado de HIV? ☐ Sim ☐ Não

23.1. Se sim, quando foi?

☐ Menos dum ano ☐ Mais dum ano ☐ Não sabe

23.2. Mudou-se de residência antes do diagnostico HIV? ☐ Sim ☐ Não ☐ Não sabe

24. Mudou-se de residência antes de começar o TARV? ☐ Sim ☐ Não ☐ Não sabe

25. Desde que iniciou seguimento nas consultas TARV, quantas vezes mudou de residência? ☐ 1-2 ☐3-4 ☐ >5 ☐ Não sabe

26. Durante a sua mudança de residência, continuou a vir as consultas TARV do Hospital Distrital da Manhiça?

☐ Sim ☐ Não ☐ Não sabe

26.1. Se não, foi feita a transferência para as consultas TARV do lugar de destino?

☐ Sim ☐ Não ☐ Não sabe

26.2. Se não foi feita a transferência, qual foi o motivo?

☐ Não informou a sua mudança de residência ☐ Pediu mas não foi aceite ☐ Não sabe

☐ outro |__|__|__|__|__|__|__|__|__|__|__|__|__|__|__|__|__|__|__|__|__|__|__|__|__|__|

|__|__|__|__|__|__|__|__|__|__|__|__|__|__|__|__|__|__|__|__|__|__|__|__|__|__|

27. Foi alguma vez as consultas HIV do lugar de destino? ☐ Sim ☐ Não ☐ Não sabe

28. Teve acesso aos ARV no lugar de destino? (Se não passa a 28.1, se sim passa a 28.2)

☐ Sim ☐ Não ☐ Não sabe

28.1. Se não, qual foi o motivo?

☐ Não procurou assistência clínica

☐ Não sabia que poderia ser seguido no lugar de destino

☐ Não foi aceite seguimento na unidade sanitária onde procurou

☐ Não havia ARV

☐ Não sabe

☐ outro |__|__|__|__|__|__|__|__|__|__|__|__|__|__|__|__|__|__|__|__|__|__|__|__|__|__|

|__|__|__|__|__|__|__|__|__|__|__|__|__|__|__|__|__|__|__|__|__|__|__|__|__|__|

28.2. Se sim, como tem acesso aos ARV no lugar de destino?

☐ Enviados pela família/ conhecido ☐ Farmácia local ☐ Comprou no mercado ou loja

☐ outro |__|__|__|__|__|__|__|__|__|__|__|__|__|__|__|__|__|__|__|__|__|__|__|__|__|__|__|

29. Durante a sua ausencia, alguém levantou os ARV para você na farmácia do Hospital Distrital da Manhiça?

☐ Sim ☐ Não ☐ Não sabe

29.1. Se alguém levantou para você, quem foi? (assinale todas aplicáveis)

☐ Parceiro/a ☐ Filho/a ☐ Pai / mãe ☐ Confidente

☐ Outro familiar ☐ Colega de GAAC ☐ Não sabe

☐ outro |__|__|__|__|__|__|__|__|__|__|__|__|__|__|__|__|

29.2. Se alguém levantou para você, como chegaram à si os ARV?

☐ Correio ☐ Intermediário ☐ outro |__|__|__|__|__|__|__|__|__|__|__|__|__|__|__|__|

29.3. Os ARV sempre chegaram a tempo? ☐ Sim ☐ Não ☐ Não sabe

29.4. O que costumava fazer quando ficava sem ARV enviados da Manhiça?

☐ Procurar os ARV na unidade sanitária do destino

☐ Comprar os ARV no lugar de destino

☐ Tomar menos comprimidos até os ARV chegar

☐ Não fazer tratamento até os ARV chegar

☐ Não sabe

☐ outro |__|__|__|__|__|__|__|__|__|__|__|__|__|__|__|__|__|__|__|__|__|__|__|__|__|__|

|__|__|__|__|__|__|__|__|__|__|__|__|__|__|__|__|__|__|__|__|__|__|__|__|__|__|

|__|__|__|__|__|__|__|__|__|__|__|__|__|__|__|__|__|__|__|__|__|__|__|__|__|__|

30. Esteve doente alguma vez no lugar de destino? ☐ Sim ☐ Não ☐ Não sabe

31. Procurou assistência clinica alguma vez no lugar de destino?

☐ Sim ☐ Não ☐ Não sabe

31.1. Si a resposta a pergunta anterior foi sim, onde foi?

☐ Urgências/Banco de Socorro ☐ Consulta TARV ☐ Consulta pré-natal ☐ Triagem

☐ Não sabe ☐ outro |__|__|__|__|__|__|__|__|__|__|__|__|__|__|__|__|

32. Esteve internado alguma vez no lugar de destino? ☐ Sim ☐ Não ☐ Não sabe

33. Quando regressa à Manhiça consegue facilmente ser atendido nas consultas TARV? ☐ Sim ☐ Não ☐ Não sabe

34. O senhor/ senhora alguma vez interrompeu o TARV? ☐ Sim ☐ Não ☐ Não sabe

34.1. Si a resposta a pergunta anterior foi sim, por quanto tempo?

☐ Menos dum mês ☐ De um à 3 meses ☐ Mais de 3 meses ☐ Não sabe

35. Alguma vez fez seguimento de CD4 ou carga viral no lugar de destino?

☐ Sim ☐ Não ☐ Não sabe

36. Se fosse possivel, gostaria de se poder comunicar com o Hospital Distrital da Manhiça por SMS no lugar de destino?

☐ Sim ☐ Não ☐ Não sabe

FACTORES SOCIAIS, FACTORES DE RISCO

37. O senhor/ senhora tem filhos? ☐ Sim ☐ Não ☐ Não sabe

37.1. Si tem filhos, quantos? |__|__|

38. O senhor/ senhora usa preservativo com o seu parceiro regular?

☐ Sempre ☐ Não ☐ Não sempre ☐ Não sabe

39. O seu parceiro sabe que você é seropositivo? ☐ Sim ☐ Não ☐ Não sabe

40. Quantos parceiros casuais teve no ultimo ano? |__|__|__|

41. Nas suas relações casuais usou preservativo?

☐ Sempre ☐ Não ☐ Não sempre ☐ Não sabe

42. O senhor/ senhora tem alguma outra relação no ponto de destino? ☐ Sim ☐ Não ☐ Não sabe

43. O senhor/ senhora tem filhos no lugar de destino? ☐ Sim ☐ Não ☐ Não sabe

44. O senhor/ senhora usa algum dos seguintes? (assinale todos aplicáveis)

☐ Álcool ☐ Tabaco ☐ Cannabis ☐ Não sabe

☐ outro |__|__|__|__|__|__|__|__|__|__|__|__|__|__|__|__|
