# Supplementary material for: Patterns of mobility and its impact on retention in care among people living with HIV in the Manhiça District, Mozambique
Source: PLoS One. 2021 May 21;16(5):e0250844. doi: 10.1371/journal.pone.0250844 (PMC8139482; doi:10.1371/journal.pone.0250844)
Supplement: S3 File — (DOCX) [file pone.0250844.s003.docx]

| 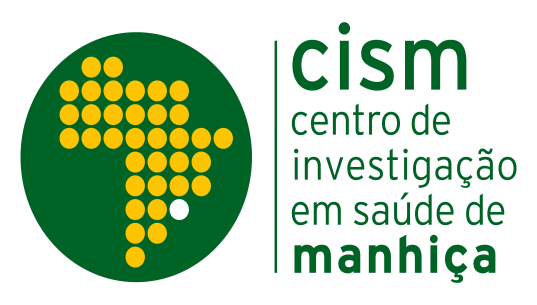 | **Study: DIASPORA**  **Survey: CONTROL_ADULT** | Serial number |
| --- | --- | --- |

|  | 1. **Does patiente lives currently in Manhiça District (DM)** ם Yes ם No 2. If participant leaves in MD, where?   □ Vila da Manhiça □ Maragra □ Palmeira/Nwamatibjana  □ Xinavane □ Maluana □ Taninga  □ Munguini □ Calanga □ 3 Fevereiro  □ Ilha Josina □ Xibukutsu □ Malavel  □ Outro \|__\|__\|__\|__\|__\|__\|__\|__\|__\|__\|__\|  2.1 If participant lives in DM,did change residency in the last 12 months?  ם Yes ם No ם Do not Know  2.2 If participant changed residency, was within Manhiça District?  ם Yes ם No ם Do notKnow  2.3 If answer for question 2.2 is No, where to?  ☐Another district within Mozambique ☐ Another Country  2.4. If changed residency for another district within the country, which one?  \|__\|__\|__\|__\|__\|__\|__\|__\|__\|__\|__\|__\|__\|__\|__\|__\|__\|__\|__\|__\|__\|__\|__\|__\|__\|__\|__\|__\|__\|  2.5. If changed to another country, which one?  ☐ South Africa ☐ Swazilând ☐ Lesotho ☐ Zimbabwe ☐ Tanzânia ☐ Botswana  ☐ Other \|__\|__\|__\|__\|__\|__\|__\|__\|__\|__\|__\|__\|__\|__\|__\| | | | | | |
| --- | --- | --- | --- | --- | --- | --- |
|  | | **Full Name** | | \|__\|__\|__\|__\|__\|__\|__\|__\|__\|__\|__\|__\|__\|__\|__\|__\|  \|__\|__\|__\|__\|__\|__\|__\|__\|__\|__\|__\|__\|__\|__\|__\|__\|  \|__\|__\|__\|__\|__\|__\|__\|__\|__\|__\|__\|__\|__\|__\|__\|__\| | | |
|  | | **Head of Household Name** | \|__\|__\|__\|__\|__\|__\|__\|__\|__\|__\|__\|__\|__\|__\|__\|__\|  \|__\|__\|__\|__\|__\|__\|__\|__\|__\|__\|__\|__\|__\|__\|__\|__\|  \|__\|__\|__\|__\|__\|__\|__\|__\|__\|__\|__\|__\|__\|__\|__\|__\| | | | |
|  | | **Neighborhood** \|__\|__\|__\|__\|__\|__\|__\|__\|__\|__\|__\|__\|__\|__\|__\|__\| | | | | |
|  | | **Birthy Date** | | | | \|__\|__\| - \|__\|__\|__\| - \|__\|__\|__\|__\| |
|  | | **Maritual Status** ☐ Married or living with a partner | | | | ☐ Separeted ☐ Widowed ☐ Single |
|  | | **Participant Perm_id** | | | \|__\|__\|__\|__\|- \|__\|__\|__\|-\|__\|__\| | |

**DIASPORA Participant Indetification**

**Control Recruted Adults**

Fulfill if patient did not change residency

HIV History

9. Remember when was diagnosed with HIV? ☐ Yes ☐ No

10. If Yes, when?

☐ Less than a year ☐ More than a year ☐ Do not Know

11. Did you ever stopped ARV treatment? ☐ Yes ☐ No ☐ Do not know

12.1. If answer for previous question was Yes, for how long?

☐ Less than a month ☐ From one to 3 months ☐ More than 3 months ☐ Do not Know

Social Factors, Risk Factors

13. Do you have childrens? ☐ Yes ☐ No ☐ Do not Know

14. If have childrens, how many? |__|__|

15. Do you use condoms with your regular partner?

☐ Always ☐ No ☐ Not always ☐ Do not Know

16. Your partner knows about you HIV status?

☐ Yes ☐ No ☐ Do not Know

17. How many casual partners had in the last year? |__|__|__|

18. In your casual relationships did you use condoms?

☐ Always ☐ No ☐ Not Always ☐ Do not Know

19. Do you use one of the followings? (check if apply)

☐ Alcohol ☐ Cigar ☐ Cannabis ☐ Do not Know

☐ Other |__|__|__|__|__|__|__|__|__|__|__|__|__|__|__|__|
