# Supplementary material for: Patterns of mobility and its impact on retention in care among people living with HIV in the Manhiça District, Mozambique
Source: PLoS One. 2021 May 21;16(5):e0250844. doi: 10.1371/journal.pone.0250844 (PMC8139482; doi:10.1371/journal.pone.0250844)
Supplement: S4 File — (DOCX) [file pone.0250844.s004.docx]

| 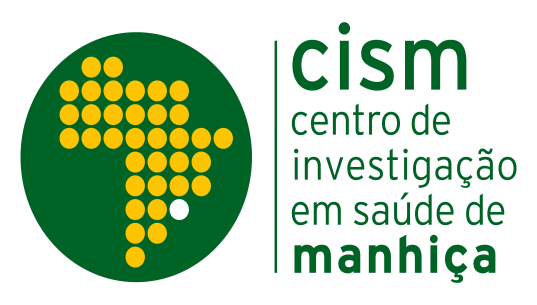 | **Estudo: DIASPORA**  **Inquérito: ADULTO_ CONTROL** | Serial number |
| --- | --- | --- |

|  | 1. Atualmente, o participante vive no Distrito da Manhiça (DM) ם Sim ם Não 2. Se o participante vive no DM, aonde?   □ Vila da Manhiça □ Maragra □ Palmeira/Nwamatibjana  □ Xinavane □ Maluana □ Taninga  □ Munguini □ Calanga □ 3 Fevereiro  □ Ilha Josina □ Xibukutsu □ Malavel  □ Outro \|__\|__\|__\|__\|__\|__\|__\|__\|__\|__\|__\|  2.1 Se o participante vive no DM, mudou de residência nos últimos 12 meses?  ם Sim ם Não ם Não sabe  2.2 Se o participante mudou de residência, foi dentro do próprio distrito da Manhiça?  ם Sim ם Não ם Não sabe  2.3 Se a pergunta 2.2 é NAO, para onde se mudou?  ☐ Outro distrito dentro de Moçambique ☐ Outro país  2.4. Si mudou para outro distrito dentro de Moçambique, em que província?  □ Cabo Delgado □ Maputo (cidade) □ Sofala  □ Gaza □ Maputo □ Tete  □ Inhambane □ Nampula □ Zambézia  □ Manica □ Niassa  2.5. Si mudou para outro país, para qual?  ☐ África do Sul ☐ Suazilândia ☐ Lesoto ☐ Zimbabwe ☐ Tanzânia ☐ Botswana  ☐ outro \|__\|__\|__\|__\|__\|__\|__\|__\|__\|__\|__\|__\|__\|__\|__\| | | | | | |
| --- | --- | --- | --- | --- | --- | --- |
|  | | **Nome completo** | | \|__\|__\|__\|__\|__\|__\|__\|__\|__\|__\|__\|__\|__\|__\|__\|__\|  \|__\|__\|__\|__\|__\|__\|__\|__\|__\|__\|__\|__\|__\|__\|__\|__\|  \|__\|__\|__\|__\|__\|__\|__\|__\|__\|__\|__\|__\|__\|__\|__\|__\| | | |
|  | | **Nome Chefe agregado** | \|__\|__\|__\|__\|__\|__\|__\|__\|__\|__\|__\|__\|__\|__\|__\|__\|  \|__\|__\|__\|__\|__\|__\|__\|__\|__\|__\|__\|__\|__\|__\|__\|__\|  \|__\|__\|__\|__\|__\|__\|__\|__\|__\|__\|__\|__\|__\|__\|__\|__\| | | | |
|  | | **Bairro em que vive** \|__\|__\|__\|__\|__\|__\|__\|__\|__\|__\|__\|__\|__\|__\|__\|__\| | | | | |
|  | | **Data de nascimento** | | | | \|__\|__\| - \|__\|__\|__\| - \|__\|__\|__\|__\| |
|  | | **Estado civil** ☐ Casado/a ou vivendo maritalmente | | | | ☐ Separado/a ☐ Viúvo/a ☐ Solteiro/a |
|  | | **Perm_id do participante** | | | \|__\|__\|__\|__\|- \|__\|__\|__\|-\|__\|__\| | |

**Identificação do participante DIASPORA**

**ADULTOS RECRUTADOS COMO CONTROLES**

À preencher se o participante NÃO mudou de residência.

HISTORIA HIV

9. Lembra-se de quando foi diagnosticado de HIV? ☐ Sim ☐ Não

10. Se sim, quando foi?

☐ Menos dum ano ☐ Mais dum ano ☐ Não sabe

11. O senhor/ senhora alguma vez interrompeu o TARV? ☐ Sim ☐ Não ☐ Não sabe

12.1. Si a resposta a pergunta anterior foi sim, por quanto tempo?

☐ Menos dum mês ☐ De um à 3 meses ☐ Mais de 3 meses ☐ Não sabe

FACTORES SOCIAIS, FACTORES DE RISCO

13. O senhor/ senhora tem filhos? ☐ Sim ☐ Não ☐ Não sabe

14. Si tem filhos, quantos? |__|__|

15. O senhor/ senhora usa preservativo com o seu parceiro regular?

☐ Sempre ☐ Não ☐ Não sempre ☐ Não sabe

16. O seu parceiro sabe que você é seropositivo? ☐ Sim ☐ Não ☐ Não sabe

17. Quantos parceiros casuais teve no ultimo ano? |__|__|__|

18. Nas suas relações casuais usou preservativo?

☐ Sempre ☐ Não ☐ Não sempre ☐ Não sabe

19. O senhor/ senhora usa algum dos seguintes? (assinale todos aplicáveis)

☐ Álcool ☐ Tabaco ☐ Cannabis ☐ Não sabe

☐ outro |__|__|__|__|__|__|__|__|__|__|__|__|__|__|__|__|
