# Supplementary material for: Patterns of mobility and its impact on retention in care among people living with HIV in the Manhiça District, Mozambique
Source: PLoS One. 2021 May 21;16(5):e0250844. doi: 10.1371/journal.pone.0250844 (PMC8139482; doi:10.1371/journal.pone.0250844)
Supplement: Attachment — Submitted filename: Response to Reviewers_final.pdf [file pone.0250844.s007.pdf]

VANDERBILT UNIVERSITY MEDICAL CENTER

Vanderbilt Institute for Global Health

February 8, 2021

PLOS ONE
Editorial Office

To the Editor:

We appreciate the time and energy the reviewers put into providing very detailed comments for improvement of our manuscript and in the editor's patience in waiting for our responses. We have done our best to respond to each of their comments. Below you will find the reviewer's or editor's comments in bold and our responses in italics. Thank you.

1. Please ensure that your manuscript meets PLOS ONE's style requirements, including those for file naming. The PLOS ONE style templates can be found at https://journals.plos.org/plosone/s/file?id=wjVg/PLOSOne_formatting_sample_main_body.pdf and https://journals.plos.org/plosone/s/file?id=ba62/PLOSOne_formatting_sample_title_authors_affiliations.pdf

2. Please provide additional details regarding participant consent. In the ethics statement in the Methods and online submission information, please ensure that you have specified (i) whether consent was informed and (ii) what type you obtained (for instance, written or verbal, and if verbal, how it was documented and witnessed). If your study included minors, state whether you obtained consent from parents or guardians. If the need for consent was waived by the ethics committee, please include this information.

Response: The following sentence has been added to the end of Ethical Considerations section of the Methods "Written informed consent was provided by all participants and approvals were obtained from the Provincial Directorate of Health".

3. In your Methods section, please provide additional information about the participant recruitment method and the demographic details of your participants. Please ensure you have provided sufficient details to replicate the analyses such as: a) a description of any inclusion/exclusion criteria that were applied to participant recruitment, and b) a statement as to whether your sample can be considered representative of a larger population."

Response: The following two sentences have been added to the Methods section, Study Design and Participants

2525 West End Avenue
Suite 750
Nashville, TN 37203

tel 615.322.9374
fax 615.343.7797
www.vumc.org/global-health

- *"Patients were included in this study if they were residents of the District of Manhiça, had migrated out of the district in the 12 months prior to study* enrollment, *and had initiated ART prior to study enrollment."*
- *"As this study recruited participants from a single district in which migratory patterns historically predominated to South Africa, our results may not be representative of migratory HIV-infected adults residing elsewhere in Mozambique or the region."*

4. Please include additional information regarding the survey or questionnaire used in the study and ensure that you have provided sufficient details that others could replicate the analyses. For instance, if you developed a questionnaire as part of this study and it is not under a copyright more restrictive than CC-BY, please include a copy, in both the original language and English, as Supporting Information.

Response: We have included the following survey questionnaire documents as Supporting information:

- *S1 File. Study Survey_Mobile adult_english*
- *S2 File. Study Survey_Mobile adult_portuguese*
- *S3 File. Study Survey_Nonmobile adult_english*
- *S4 File. Study Survey_Nonmobile adult_portuguese*

5.During our internal checks, the in-house editorial staff noted that you conducted research or obtained samples in another country. Please check the relevant national regulations and laws applying to foreign researchers and state whether you obtained the required permits and approvals. Please address this in your ethics statement in both the manuscript and submission information. In addition, please ensure that you have suitably acknowledged the contributions of any local collaborators involved in this work in your authorship list and/or Acknowledgements. Authorship criteria is based on the International Committee of Medical Journal Editors (ICMJE) Uniform Requirements for Manuscripts Submitted to Biomedical Journals - for further information please see here: https://journals.plos.org/plosone/s/authorship.

Response: Thank you. We have added to the Ethical Considerations section that approvals were obtained from the appropriate Provincial Directorate of Health. We confirm that we have suitably acknowledged contributions of our Mozambican collaborators as the first and second authors as well as authors seven and eight are local collaborators.

6. Please provide a sample size and power calculation in the Methods, or discuss the reasons for not performing one before study initiation."

Response: The following description on sample size calculation was added to the Statistical Analysis section of the Methods: "Participant recruitment was conducted in the months of December and January and based on prior clinic visit volumes it was anticipated that MDH would see approximately 100 daily patient visits during this time frame in the HIV care and treatment program and that 20% of these visits would meet eligibility criteria as a participant with history of migration out of the district (~15 patients per day or a total of 150 mobile

2525 West End Avenue
Suite 750
Nashville, TN 37203
tel 615.322.9374
fax 615.343.7797
www.vumc.org/global-health

participants). As our estimated recruitment sample was fixed (based on convenience), the statistical power to detect a difference in LTFU was variable depending on actual LTFU rates in each group (i.e for a LTFU of 20% in the non-mobile group, we would expect a 96% power to detect a difference if the LTFU was 40% in the migrant group, but only a 46% power to detect a difference if the LTFU was 30% in the migrant group)."

7. Please note that PLOS does not permit references to "data not shown." Authors should provide the relevant data within the manuscript, the Supporting Information files, or in a public repository. If the data are not a core part of the research study being presented, we ask that authors remove any references to these data.We ask that you please remove citations for unavailable and unpublished work, including manuscripts that have been submitted but not yet accepted (e.g., "unpublished work," "data not shown"). Instead, include those data as supplementary material or deposit the data in a publicly available database.

Response: Thank you. We have removed reference to not shown data from the manuscript.

8. Thank you for stating the following in the Acknowledgments Section of your manuscript:'CISM is supported by the Government of Mozambique and the Spanish Agency for International Development (AECID). ISGlobal acknowledges support from the Spanish Ministry of Science and Innovation through the "Centro de Excelencia Severo Ochoa 2019-2023" Program (CEX2018-00806-S), and support from the Generalitat de Catalunya through the CERCA Program.' We note that you have provided funding information that is not currently declared in your Funding Statement. However, funding information should not appear in the Acknowledgments section or other areas of your manuscript. We will only publish funding information present in the Funding Statement section of the online submission form. Please remove any funding-related text from the manuscript and let us know how you would like to update your Funding Statement. Currently, your Funding Statement reads as follows:'The funders had no role in the study design, data collection and analysis, decision to publish, or preparation of the manuscript'

Response: We have removed all funding information from the manuscript and updated our Funding statement

9. We note that you have indicated that data from this study are available upon request. PLOS only allows data to be available upon request if there are legal or ethical restrictions on sharing data publicly. For information on unacceptable data access restrictions, please see http://journals.plos.org/plosone/s/data-availability#loc-unacceptable-data-access-restrictions. Please include your amended statements within your cover letter; we will change the online submission form on your behalf.

Response: Thank you. We have provided a de-identified data set to accompany this manuscript as supporting information

2525 West End Avenue
Suite 750
Nashville, TN 37203

tel 615.322.9374
fax 615.343.7797
www.vumc.org/global-health

Methods, line 102: Please describe your statistical power to detect associations between your independent variables and your outcome of interest (retained in care, right?). With 195 persons in each comparison arm, are you well powered to see differences in retention in care, should they exist?

Response: Please see above, regarding alterations made related to statistical power

Methods, line 132: This is an outcome (singular) not "outcomes" (plural), right? Or am I misunderstanding something here?

Response: This has been corrected to outcome (singular)

Methods, line 143: "response" variables is what? The outcome (i.e., "retained in care" – one outcome variable?) Or did you also look at mobility? Also you may have gone between self report and pharmacy records; kindly make this more explicit.

Response: Line 143 was removed from the manuscript

Methods, line 143: do not report results in methods (i.e., move "we found no clinically or statistically significant findings" to the results section – is this what you show on line 187?)

Response: Line 143 was removed from the manuscript

Table 2: What is the "other" category that makes up more than two thirds of those didn't/couldn't get ART at travel destination? Is it for the reasons explained in the text? Consider adding this as a footer, or making more table rows to clarify.

Response: We have added the following to the sentence in the text regarding this question for better clarify however unfortunately we just do not have the reasons

Line 187 (and stat analysis in methods): is your multivariable analysis have "retention in care" as the outcome, or "self-reported perceived challenges in accessing HIV care"? Or both? Please clarify this in the methods, and do show these results, as no difference (assuming you have power to detect a difference should one exist) is an important result for all these analyses you may be doing.

Response: We have removed mention of multivariable analysis from the manuscript

Results, line 193: What's the difference between "Most (45%), stated they would prefer a 3 to 6-month dosing schedule, followed by a 3-month dosing schedule (33%),"? These two responses seem to overlap/ include each other, making interpretation of this response challenging.

Response: This sentence has been rewritten for clarity

2525 West End Avenue
Suite 750
Nashville, TN 37203

tel 615.322.9374
fax 615.343.7797
www.vumc.org/global-health

Line 201+ and Table 4: I am confused about this outcome. Why is the N for Table 4 only 349? I recommend considering a trichotomous outcome regarding retention in care: 1) retained in care, 2) Delayed ART pickups (15-60 days late) and 3) Lost to follow-up (>60 days) so that the entire cohort can be analyzed together. You are further impacting your statistical power by slicing up your study this way.
Table 4: If there is missing data (i.e., 349 vs. 390) please explain and show this so the reader can understand what's missing and where.

Response: The corresponding text in the Results section has been adapted to the following to clarify that 41 participants did not have sufficient pharmacy pick-up data to be included. Further, the footer on Table 4 was also adapted to reflect this clarification. "Forty-one participants had insufficient pharmacy pick-up data to be included in this analysis. Of the 349 participants that had complete data, 30% had at least one delay (15-60 days late) in ART pick-up documented in the pharmacy records for the 12-month period prior to survey administration, and 11% had at least one documented delay in ART pick-up of >60 days in ART. There was no significant difference in delays noted between our mobile and non-mobile cohorts (Table 4)."

Line 204: I am confused by your statement "This represents just 3% of our total population self-reporting they had ever been lost to follow-up (data not shown)." In table 4, it looks like you are showing the data: 37 report ever being lost to follow up, or 11% of the reduced cohort shown. This section is hard to follow, please revisit.

Response: Line 204 was removed from the manuscript

Discussion: please make note of how well powered your study was to detect a difference in your outcome (outcomes?), particularly in light of the fact that it appears your analysis was affected by missing data (e.g., Table 4 is not n=390)

Response: The following sentence was added to the discussion for better describing our power related to detecting a difference in LTFU between groups "This is likely due to the fact that actual LTFU rates in each group were lower than what was anticipated pre-study implementation and as a result we may not have been sufficiently powered to detect a difference."

Line 245: "However, what is striking is the large disparity between what we could document based on pharmacy pick-up records and the participants self-reported perception of their ever being lost to follow-up." Did I miss this? Where in your results do you present this disparity? This seems like an important piece of data to show. This seems like an important data point to add, perhaps to Table 4. How did this outcome vary between self-report and pharmacy records, and how did this vary across study group (mobile, not-mobile).

Response: Please see lines 207-209 and 249-253 of the track change version for changes to better clarify

2525 West End Avenue
Suite 750
Nashville, TN 37203

tel 615.322.9374
fax 615.343.7797
www.vumc.org/global-health

Line 271: Kindly remind the reader that the aforementioned study (studies? Several references are listed) are from Lesotho as the next sentence otherwise is a bit confusing.

Response: Lesotho was removed from this sentence

Abstract
Some important methodological details are not mentioned: self-reported interview data from mobile and non-mobile patients on ART care were compared with ART uptake data from clinical records, but the former comprised data from 390 participants whilst the latter was only available for 349 of these, thus possibly inviting selection bias. This fact is neither mentioned in the Abstract, nor the limitation discussed.

Response: This is mainly an issue of incomplete data in the clinical record, requiring us to exclude 41 participants from this analysis. We have added language in the Discussion section, last paragraph to better clarify.

Methods
1. The statement that 'consecutive' patients were enrolled cannot be quite correct, given the matching process used. Please clarify whether all patients reporting to be mobile were consecutively enrolled.

Response: We have added language to Study Design and Participation section to better clarify the consecutive nature of the recruitment of mobile patients first.

Regarding the matched non-mobile participants, please clarify whether they were selected from all those who met the matching criteria and presented to the clinic during the next 7 days. In other words, was the research assistant free to chose a patient who he/she preferred out of all possible matches, thus possibly inviting further selection bias, or was there a systematic rule for the selection that was followed?

Response: We have edited the language the Study Design and Participation section to clarify this question. "For each person enrolled with a history of migration, an age (± 5 years)- and sex-matched person without a history of migration, and who attended the HIV clinic was invited to participate. Controls were selected through convenience sampling from a list of patients seen at the clinic within the seven-day period following recruitment of the patient with a history of migration."

Response:
2. As mentioned for the Abstract, in the section on Data collection the information is missing that clinical records were only available from subset of 349 of the 390 participants! This needs to be explicitly stated here or latest in the Results section; and an explanation provided on why this difference occurred. This is a limitation that should also be addressed in the Discussion.

Response: Please see response provided above

3. The definition used for 'delayed ART pick-ups' (defined as gaps of more than 15 days after scheduled refills, see line 135) is rather inaccurate and broad, given that viral resistance often develops after much shorter interruptions in adherence. It would be helpful if the authors had studied whether a more stringent definition may be associated with a difference between mobile and non-mobile subgroups with respect to ART uptake.

Response: We appreciate the authors comments and recognize that yes, viral resistance does often develop with shorter interruptions in medications. However, the definitions we use here are the standard definitions for "retention in care" used across most of sub-Saharan Africa and we have added the following sentence to the end of the Definitions Utilized section "These definitions are the official definitions used by the Mozambican Ministry of Health to assess patient retention in HIV care and treatment programs.

Results

1. Some key information is missing that is required to understand the context: what is the total number of ART receiving patients registered at the Manhiça District Hospital from where the study population was selected (and of these what is the proportion of patients that achieved viral control at the time of the study)?

Response: Unfortunately, in our initial study protocol the above question was not anticipated. As such we were only allowed access to the pharmacy pick-up data of the patients who were enrolled in this study. This data is not available to us at this time.

2. What are the proportions of study participants in the two comparison groups of this study who were virally controlled at their last visit? Was there a significant difference between study groups in this respect? Data on viral control were available at the time in a data set from another province of Mozambique that I have recently seen, but if such data were not available from the Manhiça HIV care centre at the time, what were the proportions of study participants in the two groups that had a satisfactory CD4 count (say e.g. >200 cells/mm3), and did this differ between study groups? This information would be important to assess the effects of mobility on retention in ART care, and would substantially increase the value of this publication. If no such data were available at all, this would at the very least require mention in the Discussion section.

Response: Unfortunately, this data is not available to us for this study. As such we have adapted the limitations section in the discussion as requested.

3. Unfortunately there are many missing data without that this is explicitly stated (and without that the possible implications for the validity of the results are explored in the Discussion section). Examples include the following: (1) In Table 1, I was puzzled to realise that data on age was only available from 328 of 390 participants (16% missing!). How was it possible then to 'match on age'? - (2) 23% of data were missing for 'employment', yet employment features as a key variable in the consecutive analysis. – (3)

2525 West End Avenue
Suite 750
Nashville, TN 37203

tel 615.322.9374
fax 615.343.7797
www.vumc.org/global-health

Response: We are very appreciative of the reviewer catching this error on our part. Age was actually missing for only one non-mobile participant. Table 1 has been adjusted accordingly

The one and only clinical information that related to clinical severity was WHO stage, however again data were missing for 33/390 participants (8%). Note that data on clinical severity may well be associated with ART uptake. – (4) Importantly as mentioned, data on ART uptake were missing for 41/390 participants (11%) from the hospital-based data set (Table 4). Further examples of missing data can be found in tables 2 and 3. With so many data missing, one wonders how valid are the results presented? The authors should at least indicate missing data in footnotes to the various tables, mention the more important data gaps when reporting results in the text, and reflect on this issue in the Discussion section.

Response: To the extent possible we have added information into text and Table footnotes as recommended

4. In some cases, reported data do not tally within tables, or between tables and the text. Some examples: Table 1: employment data are 298, but 299 under 'type of employment'.

Response: we have reviewed the data in tables and across text and harmonized where appropriate to remove these discrepancies.

Table 2: 147 people travelled for work, but 148 of these gave information on what work they did. Also Table 2: 53 / 195 people travelled within the country (27%), but 25% are mentioned in the relevant text.

Response: We have gone back and looked at these two points. 147 persons reported the reason for travel was work, but that does not exclude persons from working if their reason for travel was listed as a different response, thus the discrepancy in numbers.

We are not seeing the 25%/27% discrepancy the reviewer mentions above and wondering if an error on their part.

Discussion
The text is generally well written but the limitations described above (e.g. due to methodological issues and missing data) should be addressed.

Response: The discussion has been adapted per the reviewer's request

Conclusion
The Conclusion section is misleading. It focuses on mobile HIV patients and the challenges that they encounter with respect to HIV care when they travel. This ignores that for the patients registered at the Manhiça Hospital the results underlying these conclusions seem to equally apply to the non-mobile group of patients. In fact, the lack of significant differences between the mobile and non-mobile groups with regards to

ART uptake is surprising and should be mentioned as an important result in the Conclusion. The real conclusion should refer to the rather worrying lack of adherence in ART uptake that affects both groups.

Response: The conclusion section has been rewritten

The paper could be substantially shortened by condensing the text. Repeat statements should be deleted (e.g. see lines 262 and 277).

Response: Line 277 was removed, thank you

Minor points
1. Please use past tense consistently across the manuscript. (Occasionally the text alternates between past and present tense).

Response: Thank you. We have edited for tense

2. Some phrases are long-winded and could be compressed to just entail the essential information.

Response: We appreciate the reviewer's comments but without examples it is difficult to know where they wish edits to be made.

3. The section on data collection and management is NOT about data analysis as suggested by its headline.

Response: We have changed the title of this section to Data Collection and Management

Please do not hesitate to contact me if any further information is necessary or would be beneficial.

Best wishes,

Troy D. Moon, MD, MPH
Associate Professor of Pediatrics,
Division of Pediatric Infectious Diseases
Vanderbilt Institute for Global Health
